# De la Prostitution dans la Ville de Paris, &c.

**Published:** 1836-10-01

**Authors:** 


### De la Prostitution dans la Ville de Paris, &c. 2 vols. 8vo. By *M. Parent Duchatelet*, M.D.

It is the duty of the medical philosopher to investigate the nature and causes of moral evil as well as of physical ills—and for this good reason, that both are inseparably connected, in causes, effects, and consequences. The investigation which occupies these two volumes has been very ably conducted by a talented physician, who paid the debt of Nature just as he had finished the work, and at the early age of 45 years! The matter of this inquiry is exceedingly curious and interesting. The inquiry could not have been effected in this country at all, because we have no police regulations or registry of abandoned females as they have in France. The labour, time, and research expended on these volumes must have been prodigious, and the work itself will stand a monument of Dr. Duchatelet's industry and perseverance.

In this number we can do little more than announce the publication, and introduce to our readers a few particulars that will create a desire for a full exposition in our next number.

We shall first notice a table which shews the number of prostitutes annually registered in Paris for 21 years—namely, from 1812 to 1832 inclusive. In the former years were registered 15523, and this astounding number has kept gradually on the increase, so that, in 1832, no less than 42,699 abandoned females were registered and licensed for the deplorable trade of destitution and destruction! A friend of the author's, while residing in London, in the year 1834, made especial enquiries as to the number of prostitutes in this capital: but nothing positive could be learnt, because no record is kept. Some magistrates estimated the number as high as seventy thousand—others as low as eighteen or twenty thousand. He thinks the truth may lie about the number of thirty or forty thousand. Still, considering the disparity of population in the two capitals, the balance is far in favour of the British metropolis. It is a curious and remarkable fact that, in the year 1812—that of the disastrous retreat from Moscow—the number of prostitutes was nearly 7000 less than two or three years afterwards.

The author has constructed a curious chart, in which the different provinces or departments are shaded deeper and deeper according to the number of prostitutes which they annually send to the metropolis. As might be expected, the departments of the Seine—that is, Paris itself, is as black as ink can make it, shewing the awful number of 4744, as its quota! One single department (Loxere) is unsullied by ink, having contributed nothing to the mass of Parisian prostitution.

There is a table indicating the avocations of the parents, and another denoting the employments of the prostitutes themselves; but from these tables we cannot collect much that would be satisfactory. This, however, may be observed, that the more sedentary the avocations—the more uncertain the employment—and the more congregated the *employées*, the more likely they are to afford nutriment to the destructive leaven of prostitution.

In Paris, the great mass of *filles publiques* emanated from the artizans. Very few were furnished by families of condition. In the provinces, a considerable number were furnished by respectable shop-keepers and professional people, as barristers, physicians, &c. But still it was from the workshops (atteliers) that the great mass of abandoned females came.

*Education.* This was difficult to ascertain ; but, in general, it was found to be very low among these unfortunate beings. One-third of them were incapable of writing their own names.

*Illegitimate Births.* An idea has prevailed that a great proportion of the women of the town were, originally, illegitimates. A rigorous investigation has disproved this opinion. The proportion among them was about 1 in 4 in Paris, but much less in the departments. It is curious that among the registered street-walkers were individuals possessed of rentes to the amount of 500 francs, and even 2000 francs annually !

*Age.* Where this could be ascertained, it was found to vary from the age of 12 years to 50. There were very few below 16, and few after the age of 40. Some, however, appear to have been allowed to inscribe their name at the age of 10 and 11—a scandalous permission !

*Primary Causes of Prostitution.* These are numerous, and probably some of those alleged are fictitious. They were as follows. Almost all of the prostituted acknowledge that they had led a disorderly or irregular life for some time before becoming actually prostitutes on the town. Few or none came to register their names, who were not deflowered previously.

*Idleness.* Among the primary and predominant causes was IDLENESS! Females abandoned by their parents, and without any avocation, see nothing but starvation before them, and are therefore fain to fly to prostitution for support! Many who came to inscribe their names on the fatal list, had not tasted food for some days before they could make up their minds to the dire alternative!

VANITY. This, and the wish to dress finely, added greatly to idleness, as a cause of prostitution, especially in Paris.

SEDUCTION. This is a very efficient cause, particularly in the country. The poor deceived creatures come to Paris in search of their seducers, and finding themselves without support, are obliged to have recourse to the town. There are always a great number of abominable bawds on the watch for these country girls. Many who are seduced in the country, come also to Paris, to hide their shame, and with the full intention of going on the town.

*Domestic Chagrin and bad Treatment*, at home, drive many into this wretched line of life. The brutality of step-fathers and step-mothers is one of the most common excuses which females from the country make, when they first inscribe.

*Long Sojourn in Hospitals* is set down among the causes of prostitution. In these institutions the bawds have their agents always on the watch to seduce young females into the haunts of ill-fame on leaving the establishment.

*The Misconduct and bad Example of Parents* are no inefficient causes of loss of virtue. Widowers living openly with concubines afford a sad example to the daughters, and lead to the most disastrous consequences. Separation of fathers and mothers has a similar effect.

*Loss of Employment and Want* are, of all causes, the most accused, and perhaps justly so, by those who inscribe their names on the black list of prostitution! The salaries of females employed in dress-work, and numerous other sedentary avocations, are only sufficient to procure subsistence for the time, and when the season is over, or any circumstance throws them out of work, they are forced to go on the town, or starve. These causes are daily becoming more efficient, by the multitudes of young men who are now usurping the habits and employments of females in all kinds of work. It is often found that mothers abandoned by their husbands have taken to prostitution for the support of their children—and girls for the support of their aged parents!

Our author observes that, at first sight, one would be tempted to attribute much of modern prostitution to modern civilization; but history informs us that this vice was very common in the middle ages, and consequently in the midst of barbarism. Our acquaintance also with people in a state of incivilization leads to the inevitable conclusion that prostitution is, as it were, inherent in human nature!

The following numerical particulars are curious and melancholy.

Out of 5183 inscriptions, there were 164 instances of two sisters inscribing at the same time—4 instances of three sisters inscribing—3 instances of four sisters entering at once on this wretched system of life. There were 16 instances of mother and daughter inscribing together—4 of aunt and niece—22 of cousins coming to the same office for the same purpose!

### Chap. II.—Manners and Habits of Prostitutes.

This enquiry is considered by our author as of the utmost importance; because we cannot expect to introduce any amelioration among these unfortunates, without a knowledge of their manners and habits.

If we only take a superficial view of these "filles de joie" (improperly so called), we might be led to conclude that they delight in their profession, and are more than usually happy. But when they are in prisons or hospitals, where they have leisure for reflection, and speak their sentiments, they present a very different aspect. They then afford incontestible proofs of the misery which presses on their souls at the moment they are forced to wear the semblance of happiness! They are conscious of their depravity and degradation, and, consequently, they are uneasy except when among the companions of their own ignominy and disgrace. The sight of modest women and of the mothers of families is insupportable, and, by a kind of involuntary impulse, they are inclined to insult them, in vengeance for the degraded state in which they find themselves! However immodest and indecent they may be in their vocation, they endeaaour to hide their disgrace under other circumstances, and endeavour, where it is possible, to pass for what they are not. In general, they are dreadfully afraid of meeting those who knew them before they became abandoned. The sight of a juvenile acquaintance, in an hospital, caused one of these unfortunates to become insane! M. Pariset, indeed, has seen numerous instances of prostitutes becoming insane, from reflection on their wretched and despicable state. At one time, in La Pitié, there was no chapel for the impure wards. Afterwards an altar was erected for their use. They were overwhelmed with joy on the occasion—not from religious feelings, but because they considered themselves as not so much degraded in the eyes of society as they had been before the altar was erected.

*Religious Sentiments.* The Parisian prostitutes are, in general, profoundly ignorant of all religious duties, and destitute of religious feelings. A great number of them are scarcely impressed with the idea of a Divine Providence! Nevertheless many of them, by a kind of instinct, are taken with devotional feelings at the sight of funerals, or when on the bed of death.

*Prostitutes still preserve some Sense of Modesty.* Many instances of this kind are given by our author, all of which prove, that there is much less of this remaining amongst them than amongst the same class in this country. There is a great improvement in the external behaviour of the Parisian prostitutes within these last few years. The same may be said of the same class in this country. Those who remember the streets of London 40 years ago, and compare 1796 with 1835, must be able to testify to the immense amelioration of external morals in that short space of time!

*Marks on the Bodies of Prostitutes.* Many of these unhappy creatures have letters and characters imprinted on their bodies, in the same fashion as our sailors and soldiers have. They are generally the proper names of men or women, followed by the letters P. L. V., *pour la vie.* It is not a little curious that, on the bodies of young females, we find the names of men—while, on those of a certain age, we find the names of females. In this latter case, the engraving (if we may use the term) is almost always between the umbilicus and the pubes. Although "pour la vie" is attached to the first, and even many subsequent names, yet it often happens that twenty, thirty, or forty new lovers, "for life," are seen engraved on the frail and false tombstone! Of late years, they have found the means of effacing an old inscription, when they wish to insert the name of a new lover. Sulphuric acid and indigo are said to afford the means of effacing the old marks.

*Intemperance.* This is almost universal, even among the French prostitutes—and it is not much to be wondered at in any country. The life of a prostitute is one of extremes—all excitement at one time—all torpor, ennui, or triste reflection at another. It is natural to expect that they would fly to stimulants in the moments of sadness—and the habit, being once formed, is never afterwards shaken off. The habit of *malpropreté* and mendacity is also nearly universal.

*Good Qualities.* There is hardly any class of people, however debased and depraved, among whom some favourable traits may not be discovered. Prostitutes form no exception. One good quality consists in their universal disposition to assist each other in sickness, misfortune, or distress. If one of their comrades is taken ill, they all hasten to afford her succour. They convey her to the hospital, if necessary, and there visit her daily afterwards. If put in prison, they supply her with clothes, and every kind of comfort in their power. They will strip themselves, rather than see their comrade naked! This generous sentiment is probably engendered by the conviction, that they are a class abandoned and despised by the world, and, consequently, that they have nothing to hope but from each other. But it is not a little remarkable, that this sympathy for the sufferings of their own class not unfrequently extends beyond the circle, and reaches those who do not belong to the same vocation. Many instances of this kind are related by our author, and which are exceedingly honorable to the hearts of these unfortunate creatures! Nothing is more common than to find prostitutes supporting aged parents, or distressed sisters, out of the miserable earnings of their wretched trade! It has been sufficiently ascertained, that prostitutes are far from being anxious to avoid pregnancy. On the contrary, they rather feel proud of this state. They are sure of succour from their companions, before, during, and subsequent to parturition. They make excellent mothers and nurses—often much better, indeed, than are found among those who are not reduced to a state of prostitution.

*Almost every Prostitute has a particular Favourite.* Whatever be the violence of passion that has led young females into a state of prostitution, nothing can be more certain than that they soon get entirely indifferent to the illicit and promiscuous intercourse with the other sex—which indifference often amounts to a loathing, which they are necessarily obliged to conceal, and even disguise. The practice of selecting some favourite, to whom they become attached for a time, is almost universal. The letters addressed to them, and which are often opened by the police, especially when they get into prison, disclose the names of statesmen, generals, literati—all ranks, from the Crown downwards! Prostitutes of the better classes, however, generally select their favourites from among students of law and medicine, and also among young advocates. The middle classes of *filles publiques* find their paramours among young shopkeepers of every description, especially the tailors and habit-makers, a genus so exceedingly numerous in Paris. To these may be added the hair-dressers, ambulant musicians, &c. &c. The lower orders of the meretricious tribe attach themselves to the working people (ouvriers) of every description and grade, down to a class of the most abandoned villains of the human species, in the form of man. One of the most remarkable traits in the prostitute character, is their extreme attachment to their paramours. They not only take every pains to save them from the offended laws, but even support them when out of employ. "*A considerable number of young men in Paris have no other means of subsistence, than the wages of prostitution earned by these filles publiques.*" Whenever these females enter a house kept by a prostitute proprietor, they stipulate for the free entrance of their favourites twice, thrice, or four times a week. Among the lowest classes of prostitutes, the most inhuman treatment on the part of their flash men will not cause them to rebel, or part from their tyrant masters. They are often brought to the hospitals beaten to mummies, by these devils in the shape of men; but their victims will never bring them to punishment! These fellows, indeed, are almost all robbers, thieves, and rascals of the blackest dye.

The author enters into a long detail of the most deplorable, digusting vice, into which a considerable number of the Parisian prostitutes fall, and which we cannot here describe. It is sufficient to say, that it is an unnatural intersexual connexion with each other! ! The details are too horrible to be placed on record!

*Classification of Prostitutes.* Hitherto our author has spoken only of these abandoned creatures *en masse.* But there is a necessity for dividing them into classes. There are two or three genera, the most dangerous to society and destructive to morals—who are, in reality, as complete strumpets as any on the pavé of Paris, who yet defy the police, and even bring legal actions against those who may incautiously designate them as prostitutes. We shall glance at some of these—unfortunately not peculiar to the Gallic metropolis.

*Femmes Gallantes.* These are the tip-top of their profession—the aristocracy of the ladies of easy virtue. They are generally kept-mistresses, or partly so. They dress in the highest style of fashion—live in unbounded luxury and extravagance (as long at least as they can)—and demure themselves in all public places as modestly as "a —— at a christening." Yet there is something in their air and tone that clearly indicates them to all who are in pursuit of such objects. They suffer themselves to be accosted—to be followed—and they soon lead their admirers to perdition! They are known by the term "*femmes gallantes;*" but we are, happily, unable to translate this term into our own language.

*Femmes à Parties.* We know of no English designation corresponding with this term. Beauty alone is not sufficient for them. They add the charm of various accomplishments and graces to an "*esprit cultivé.*" Admission to their circles must be through an introduction of a *respectable* friend. They have soirées—give elegant entertainments—and the gaming-table forms a part of the evening's amusement and ruin! They are, of course, *a l'abris de la police.*

*Femmes de Spectacle et de Theatres.* This is a very numerous sisterhood. Their manners and habitudes differ from the preceding classes or genera. They muster from four to five hundred in Paris. These three divisions, as we said before, are veritable prostitutes, though not registered, or under the surveillance of the police. They are the most dangerous of all—and are the ruin of many thousands annually in the French metropolis.

*Filles en Carte.* These are they who are registered and licensed by the police. They are subjected to periodical examinations by authorised medical inspectors. But even in this grand class there are numerous grades, the higher looking down on the lower orders with the most sovereign contempt. They have their *habitats,* and dare not, or cannot, pass the barriers which custom and their own wills have erected. Some of the most beautiful creatures in existence, make their debut in the most infamous haunts, and there grow old or die, without the power of passing into a higher order of meretricious society! The reason is, they contract the habits and manners of the men with whom they cohabit, and soon become unfit for cohabitation with those of a better grade. The same rule prevents prostitutes from degenerating or mixing with a class inferior to those with whom they have been, for some time, associated.

We pass over a number of short sections on the classes and distinctions of prostitutes, in order to approach subjects that bear more directly on our own science—namely, physical and physiological considerations.

### Chap. III.—Physiological Considerations.

*Corpulency.* The embonpoint and brilliant health of a great portion of prostitutes strike the observer with surprize, when he sees them collected in masses at any public place. The exceptions, however, are numerous, and we observe a certain sprinkling of individuals meagre, or even emaciated. Those who live constantly among these unfortunates, and have them always under their eyes, remark that this corpulency seldom commences till after the age of 25 or 30 years. It is rarely seen among the young, or those who have been but a short time on the town. Many people, including physicians, have attributed this corpulency to the frequent use of mercury—the fact being certain that people generally become fleshy and healthy after a course of that medicine. But the physicians of dispensaries, prisons, and hospitals deny this position, averring that they see great numbers of prostitutes become embonpoint, who have not had any venereal disease for years previously. These and our author attribute the phenomenon to inactivity of life, rich food, and frequent use of the warm-bath. Prostitutes are almost always eating and drinking—lie in bed till ten or eleven o'clock in the day. The meagre and emaciated exceptions may be accounted for by poverty and hunger—or constitutional dispositions.

*Peculiar Alteration of the Voice.* We frequently observe prostitutes who are most beautiful in face and figure—elegant in their manners, and even refined in their tastes—in short, who seem the beau ideal of Venus herself—till they open their mouths—when, instead of musical tones and silver sounds, they utter such hoarse and discordant intonations as crucify the ears of the listeners! This vox rauca occurs about the age of 25 years—and is very general, though it is more common amongst the lower than the higher orders of the sisterhood—amongst those who linger about the doors of cabarets—who often get drunk, and then indulge in loud vociferations. The true causes may be inferred from these observations, namely, the application of cold and damp, when lightly clothed—intemperance—and great exertion of the voice. Some causes have been assigned, which are either fanciful or revolting, and which we pass over. This vox rauca has been on the decline in Paris since the police regulations have become more strict.

Two sections are devoted to the colour of the hair, eyes, and eye-brows of prostitutes—and also to the stature; but we do not deem it necessary to notice them here.

*State of the Genital Organs.* An immense experience in the examination of prostitutes has made our author very doubtful and timid as to the proofs of virginity, and also of defloration. He give a curious history of two young girls who were insulted in the street by some young men, and reproached with prostitution. They brought their accusers before the magistrate, and as the young men persisted in the charge, they offered to submit to an examination as to their virginity. A most able physician undertook the investigation. After a most careful examination, he deposed that, in respect to one of the girls he could not determine whether she was or was not a virgin. As to the other, he suspected that she was not a virgin; but he could not swear to it! It was afterwards ascertained that they were common girls of the town, and that one of them had been twice, or thrice in hospital for syphilis!

This circumstance made a deep impression on our author at the time, and he lost no opportunity afterwards of investigating the subject. As we before hinted, the more he sought to ascertain the marks of defloration and the proofs of virginity, the more difficult he found the inquiry, and the more diffident he became. In fact, he acknowledges that, on the verbal declarations of young girls, he could often depend far more than on actual inspection of the genital organs. However, our author set to work a great number of physicians and surgeons, who had extensive opportunities of examining the organs of generation in prostitutes, and the result is as follows:—The genital parts do not present any particular alteration or appearance in these females, different from married and modest women. The constant employment of the speculum, of late years, has rendered the enquiry more positive in its results. It was frequently found that young girls, who had very recently become prostitutes, had vaginas as large as women who had borne several children—and, on the other hand, they often examined women, who had been 12 or 15 years on the town, and who evinced signs of premature decrepitude, and yet where the vagina and other parts of the genitals presented nothing at all remarkable. They examined a woman, aged 51 years, at the Prison of Madelonnettes, who had been on the town since the age of 15, and yet the genital organs might have passed for those of a maid just turned of eighteen years.

These facts, which have been accumulated and verified on the largest scale, present important subjects for reflection to the medico-legal inquirer. Works on medical jurisprudence gives us such minute rules for ascertaining the fact of a rape having been committed, that nothing seems more easy than to prove the truth or falsehood of the indictment. Yet physicians and surgeons of the most unbounded experience, and who are in the habit of daily making personal examinations of females, are so bewildered with doubt, that they hardly ever give a positive opinion on the subjects of violation or virginity. When we consider how often these decisions are left to a jury of MATRONS, we may well shudder at the results!

M. Jacquemin has discovered a new sign of pregnancy by the examination of prostitutes. It is a deep violet tint, sometimes like the lees of wine, which the whole mucous membrane of the vagina presents, when utero-gestation is going on. This sign has never failed him, and he relies on it with the most implicit confidence. This chapter concludes with some observations on the state of the clitoris, the anus, and the function of menstruation. Nothing very decisive, however, can be concluded from examination on these points.

We have brought our analysis to the end of the first half of the first volume. In our next Number, we shall give a long article from the same work, which is one of the most curious and important that has issued from the press for some years.

